# Supplementary material for: Video-based interventions to improve self-assessment accuracy among physicians: A systematic review
Source: PLoS One. 2023 Jul 13;18(7):e0288474. doi: 10.1371/journal.pone.0288474 (PMC10343035; doi:10.1371/journal.pone.0288474)

**Supplement 1.** Search strategy

**Search limits:** Case reports, Comments, editorial, conference proceedings are removed

Summary of results per database (pre-deduplication)

| **Database** | **2022 Hits** | **2021 Hits** | **2020 Hits** | **2019 Hits** | **Total Hits** |
| --- | --- | --- | --- | --- | --- |
| Medline | 68 | 97 | 35 | 477 | 677 |
| Embase | 71 | 67 | 41 | 323 | 502 |
| EBM Reviews | 5 | 193 | 47 | 254 | 494 |
| Scopus | 55 | 52 | 156 | 440 | 703 |
| **Total hits** | **199** | **409** | **279** | **1494** | **2376** |

# Medline

Database(s): **Ovid MEDLINE: Epub Ahead of Print, In-Process & Other Non-Indexed Citations, Ovid MEDLINE® Daily and Ovid MEDLINE®**1946-Present
Search Strategy:

| **#** | **Searches** | **Results** |
| --- | --- | --- |
| 1 | Self-Assessment/ | 13198 |
| 2 | Self-Evaluation Programs/ | 964 |
| 3 | Self Report/ | 41166 |
| 4 | self efficacy/ | 23459 |
| 5 | self-directed learning as topic/ | 104 |
| 6 | ((Self or personal*) adj3 (measure* or assess* or evaluat* or reflect* or rating* or rate* or critique* or criticism* or apprais* or audit* or validation* or review* or score* or scoring or grade* or grading* or efficacy or feedback or examin* or learn* or report* or direct* or confiden* or perception* or aware* or performance* or judg* or debrief* or observ*)).ab,kf,ti. | 382961 |
| 7 | (own adj2 (measure* or assess* or evaluat* or reflect* or rating* or rate* or critique* or criticism* or apprais* or audit* or validation* or review* or score* or scoring or grade* or grading* or efficacy or feedback or examin* or learn* or report* or direct* or confiden* or perception* or aware* or performance* or judg* or debrief* or observ*)).ab,kf,ti. | 12140 |
| 8 | or/1-7 | 415370 |
| 9 | Videotape Recording/ or Video recording/ | 38983 |
| 10 | (video* adj2 (measure* or assess* or evaluat* or reflect* or rating* or rate* or critique* or criticism* or apprais* or audit* or validation* or review* or score* or scoring or grade* or grading* or efficacy or feedback or examin* or learn* or report* or direct* or confiden* or perception* or aware* or performance* or judg* or debrief* or observ*)).ab,kf,ti. | 18094 |
| 11 | 9 or 10 | 52185 |
| 12 | "Internship and Residency"/ | 57853 |
| 13 | education, medical, graduate/ or education, medical, continuing/ or teaching rounds/ | 57726 |
| 14 | exp Physicians/ | 168762 |
| 15 | exp Surgeons/ | 14838 |
| 16 | (post?graduate* adj3 medic*).ab,kf,ti. | 5175 |
| 17 | (Clinician* or Physician* or doctor* or house officer* or registrar* or Allergist* or immunologist* or Anesthesiologist* or Anesthetist* or Cardiologist* or Dermatologist* or Endocrinologist* or Gastroenterologist* or General Practitioner* or Geriatrician* or Nephrologist* or Neurologist* or Oncologist* or Ophthalmologist* or Osteopathi* or Otolaryngologist* or Pathologist* or Pediatrician* or Peadiatrician* or Physiatrist* or Pulmonologist* or Radiologist* or Rheumatologist* or Surgeon* or Urologist* or Neuro?surgeon* or Orthopedic* or endoscopist* or gynecologist* or Obstetric* or Ophthalmolog* or Orthognathic* or Otolaryngolog* or Neurotolog* or Traumatolog* or Urolog* or PGY* or h?ematologist* or hepatologist* or neonatologist* or pulmonologist*).ab,kf,ti. | 1552428 |
| 18 | ((residen* or fellow* or trainee*) adj2 (medic* or clinical)).ab,kf,ti. | 19052 |
| 19 | or/12-18 | 1663313 |
| 20 | 8 and 11 and 19 | 634 |
| 21 | (case reports or comment or editorial).pt. | 3667790 |
| 22 | 20 not 21 | 628 |
| 23 | ("20190917" or "20190918" or "20190919" or 2019092* or 2019093* or 2020*).dt,ez,da. | 2161438 |
| 24 | 22 and 23 | 75 |
| 25 | (20191* or 202008* or 202009* or 20201* or 2021*).dt,ez,da. | 3379094 |
| 26 | 22 and 25 | 128 |
| 27 | (2021082* or 2021083* or 202109* or 202110* or 202111* or 202112* or 2022*).dt,ez,da. | 2098116 |
| 28 | 22 and 27 | 68 |

Copy of a live search [VPN must be connected]

<https://myaccess.library.utoronto.ca/login?url=http://ovidsp.ovid.com/ovidweb.cgi?T=JS&NEWS=N&PAGE=main&SHAREDSEARCHID=3gLRvjzDn1ozlmAF7wT5RXfIWPWj7DhRTwTrn0u9KM5dqhLLzXkDGfwEieaD4SAKN>

# EMBASE

Database(s): **Embase Classic+Embase**1947 to 2022 August 22
Search Strategy:

| **#** | **Searches** | **Results** |
| --- | --- | --- |
| 1 | self evaluation/ | 35758 |
| 2 | self report/ | 142250 |
| 3 | self concept/ | 104657 |
| 4 | self-directed learning/ | 1324 |
| 5 | ((Self or personal*) adj3 (measure* or assess* or evaluat* or reflect* or rating* or rate* or critique* or criticism* or apprais* or audit* or validation* or review* or score* or scoring or grade* or grading* or efficacy or feedback or examin* or learn* or report* or direct* or confiden* or perception* or aware* or performance* or judg* or debrief* or observ*)).ab,kw,ti. | 497982 |
| 6 | (own adj2 (measure* or assess* or evaluat* or reflect* or rating* or rate* or critique* or criticism* or apprais* or audit* or validation* or review* or score* or scoring or grade* or grading* or efficacy or feedback or examin* or learn* or report* or direct* or confiden* or perception* or aware* or performance* or judg* or debrief* or observ*)).ab,kw,ti. | 16782 |
| 7 | or/1-6 | 605793 |
| 8 | *videotape/ | 299 |
| 9 | *videorecording/ or audiovisual recording/ | 19353 |
| 10 | (video* adj2 (measure* or assess* or evaluat* or reflect* or rating* or rate* or critique* or criticism* or apprais* or audit* or validation* or review* or score* or scoring or grade* or grading* or efficacy or feedback or examin* or learn* or report* or direct* or confiden* or perception* or aware* or performance* or judg* or debrief* or observ*)).ab,kw,ti. | 25528 |
| 11 | 8 or 9 or 10 | 41682 |
| 12 | residency education/ or surgical training/ or teaching round/ | 52710 |
| 13 | exp physician/ | 933589 |
| 14 | (post?graduate* adj3 medic*).ab,kw,ti. | 6662 |
| 15 | (Clinician* or Physician* or doctor* or house officer* or registrar* or Allergist* or immunologist* or Anesthesiologist* or Anesthetist* or Cardiologist* or Dermatologist* or Endocrinologist* or Gastroenterologist* or General Practitioner* or Geriatrician* or Nephrologist* or Neurologist* or Oncologist* or Ophthalmologist* or Osteopathi* or Otolaryngologist* or Pathologist* or Pediatrician* or Peadiatrician* or Physiatrist* or Pulmonologist* or Radiologist* or Rheumatologist* or Surgeon* or Urologist* or Neuro?surgeon* or Orthopedic* or endoscopist* or gynecologist* or Obstetric* or Ophthalmolog* or Orthognathic* or Otolaryngolog* or Neurotolog* or Traumatolog* or Urolog* or PGY* or h?ematologist* or hepatologist* or neonatologist* or pulmonologist*).ab,kw,ti. | 2363542 |
| 16 | ((residen* or fellow* or trainee*) adj2 (medic* or clinical)).ab,kw,ti. | 32447 |
| 17 | 12 or 13 or 14 or 15 or 16 | 2644425 |
| 18 | 7 and 11 and 17 | 746 |
| 19 | (conference abstract or conference paper or conference review or editorial or letter or book review).pt. | 7254854 |
| 20 | 18 not 19 | 451 |
| 21 | exp animal/ not human/ | 5934441 |
| 22 | 20 not 21 | 447 |
| 23 | limit 22 to dc=20210820-20220823 | 71 |

A link to the live search [VPN must be connected]

<https://myaccess.library.utoronto.ca/login?url=http://ovidsp.ovid.com/ovidweb.cgi?T=JS&NEWS=N&PAGE=main&SHAREDSEARCHID=4WG1zw95T4oCwhnsEzA4pxowsnWOVBOTm7NjD0LaQbuvklqCRsht4oAVmEKiNsNwv>

# Scopus

## ( TITLE-ABS-KEY ( ( self  OR  personal* )  W/3  ( measure*  OR  assess*  OR  evaluat*  OR  reflect*  OR  rating*  OR  rate*  OR  critique*  OR  criticism*  OR  apprais*  OR  audit*  OR  validation*  OR  review*  OR  score*  OR  scoring  OR  grade*  OR  grading*  OR  efficacy  OR  feedback  OR  examin*  OR  learn*  OR  report*  OR  direct*  OR  confiden*  OR  perception*  OR  aware*  OR  performance*  OR  judg*  OR  debrief*  OR  observ* ) ) )  AND  ( TITLE-ABS-KEY ( video*  W/2  ( measure*  OR  assess*  OR  evaluat*  OR  reflect*  OR  rating*  OR  rate*  OR  critique*  OR  criticism*  OR  apprais*  OR  audit*  OR  validation*  OR  review*  OR  score*  OR  scoring  OR  grade*  OR  grading*  OR  efficacy  OR  feedback  OR  examin*  OR  learn*  OR  report*  OR  direct*  OR  confiden*  OR  perception*  OR  aware*  OR  performance*  OR  judg*  OR  debrief*  OR  observ* ) ) )  AND  ( ( TITLE-ABS ( ( post-graduate*  OR  postgraduate*  OR  residen*  OR  fellow*  OR  trainee* )  W/3  ( medic*  OR  clinical ) ) )  OR  ( TITLE-ABS ( ( clinician*  OR  physician*  OR  doctor*  OR  "house officer*"  OR  registrar*  OR  allergist*  OR  immunologist*  OR  anesthesiologist*  OR  anesthetist*  OR  cardiologist*  OR  dermatologist*  OR  endocrinologist*  OR  gastroenterologist*  OR  "general practitioner*"  OR  geriatrician*  OR  nephrologist*  OR  neurologist*  OR  oncologist*  OR  ophthalmologist*  OR  osteopathi*  OR  otolaryngologist*  OR  pathologist*  OR  pediatrician*  OR  peadiatrician*  OR  physiatrist*  OR  pulmonologist*  OR  radiologist*  OR  rheumatologist*  OR  surgeon*  OR  urologist*  OR  neuro?surgeon*  OR  orthopedic*  OR  endoscopist*  OR  gynecologist*  OR  obstetric*  OR  ophthalmolog*  OR  orthognathic*  OR  otolaryngolog*  OR  neurotolog*  OR  traumatolog*  OR  urolog*  OR  pgy*  OR  h?ematologist*  OR  hepatologist*  OR  neonatologist*  OR  pulmonologist* ) ) ) )  AND  ( ORIG-LOAD-DATE  >  20210820 )


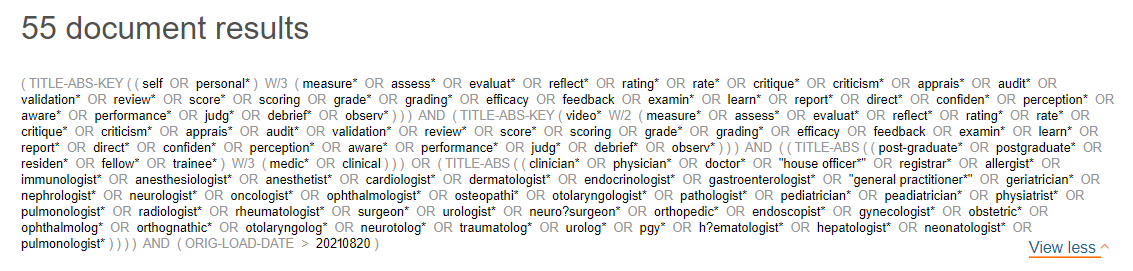

Supplement: S1 File — (DOCX) [file pone.0288474.s002.docx]
