# Supplementary material for: Video-based interventions to improve self-assessment accuracy among physicians: A systematic review
Source: PLoS One. 2023 Jul 13;18(7):e0288474. doi: 10.1371/journal.pone.0288474 (PMC10343035; doi:10.1371/journal.pone.0288474)
Supplement: S2 File — (DOCX) [file pone.0288474.s003.docx]

**Supplement 3.** QUADAS-2 tool modified for self-assessment accuracy studies

| Domain 1: Participant Selection | |
| --- | --- |
| 1. Risk of Bias | |
| Describe methods of participant selection: | |
| Was a consecutive of random sample of participants enrolled?  Was a case-control design avoided?  Did the study avoid inappropriate exclusions? | Yes/ No/ Unclear  Yes/ No/ Unclear  Yes/ No/ Unclear |
| Could the selection of participants have introduced bias? | Risk: Low/ High/ Unclear |
| 1. Concerns regarding applicability | |
| Describe included participants (prior testing, presentation, intended use of self-assessment and setting): | |
| Is there concern that the included participants do not match the review question? | Concern: Low/ High/ Unclear |

| Domain 2: Self-assessment(s) | |
| --- | --- |
| 1. Risk of Bias | |
| Describe the self-assessment and how it was conducted and interpreted: | |
| Were the self-assessment results interpreted without knowledge of the results of the external assessment?  If a threshold was used, was it pre-specified? | Yes/ No/ Unclear  Yes/ No/ Unclear |
| Could the conduct of interpretation of the self-assessment have introduced bias? | Risk: Low/ High/ Unclear |
| 1. Concerns regarding applicability | |
| Is there concern that the self-assessment, its conduct, or interpretation differ from the review question? | Concern: Low/ High/ Unclear |

| Domain 3: External Assessment | |
| --- | --- |
| 1. Risk of Bias | |
| Describe the external assessment and how it was conducted and interpreted: | |
| Is the external assessment likely to correctly classify the targeted skill(s)?  Were the external assessment results interpreted without knowledge of the results of the self-assessment? | Yes/ No/ Unclear  Yes/ No/ Unclear |
| Could the external assessment, its conduct, or its interpretation have introduced bias? | Risk: Low/ High/ Unclear |
| 1. Concerns regarding applicability | |
| Is there concern that the targeted skill(s) as defined by the external assessment does not match the review question? | Concern: Low/ High/ Unclear |

| Domain 4: Flow and Timing | |
| --- | --- |
| 1. Risk of Bias | |
| Describe any participants who did were not included in the self-assessment(s) and/or external assessment who were excluded: | |
| Describe the time interval and any interventions between self-assessment(s) and external assessment: | |
| Was there an appropriate interval between self-assessment(s)?  Were all participants assessed using external assessment?  Were all participants assessed using the same external assessment?  Were all participants included in the analysis? | Yes/ No/ Unclear  Yes/ No/ Unclear  Yes/ No/ Unclear  Yes/ No/ Unclear |
| Could the participant flow have introduced bias? | Risk: Low/ High/ Unclear |
