# Supplementary material for: Video-based interventions to improve self-assessment accuracy among physicians: A systematic review
Source: PLoS One. 2023 Jul 13;18(7):e0288474. doi: 10.1371/journal.pone.0288474 (PMC10343035; doi:10.1371/journal.pone.0288474)
Supplement: S3 File — (DOCX) [file pone.0288474.s004.docx]

**Supplement 2.** Reference list for excluded studies from full-text review, with respective rationale for exclusion

1. Ghoneim N, Dariya V, Guffey D, et al. Teaching NICU Fellows How to Relay Difficult News Using a Simulation-Based Curriculum: Does Comfort Lead to Competence?. *Teach Learn Med*. 2019;31(2):207-221. doi:10.1080/10401334.2018.1490649; Excluded because there was no analysis of self-assessment accuracy
2. Brewster LP, Risucci DA, Joehl RJ, et al. Comparison of resident self-assessments with trained faculty and standardized patient assessments of clinical and technical skills in a structured educational module. *Am J Surg*. 2008;195(1):1-4. doi:10.1016/j.amjsurg.2007.08.048. Excluded because there was no intervention used to improve self-assessment accuracy
3. Weller JM, Robinson BJ, Jolly B, et al. Psychometric characteristics of simulation-based assessment in anaesthesia and accuracy of self-assessed scores. *Anaesthesia*. 2005;60(3):245-250. doi:10.1111/j.1365-2044.2004.04073.x. Excluded because the video review was not used for improving self-assessment accuracy
4. Jethwa AR, Perdoni CJ, Kelly EA, Yueh B, Levine SC, Adams ME. Randomized Controlled Pilot Study of Video Self-assessment for Resident Mastoidectomy Training. *OTO Open*. 2018;2(2):2473974X18770417. Published 2018 Apr 12. doi:10.1177/2473974X18770417. Excluded because the improvement in self-assessment accuracy was not reported
5. Hu Y, Tiemann D, Michael Brunt L. Video self-assessment of basic suturing and knot tying skills by novice trainees. *J Surg Educ*. 2013;70(2):279-283. doi:10.1016/j.jsurg.2012.10.003. Excluded because there was no comparison of self-assessment accuracy with and without video-based intervention
6. Jamshidi R, LaMasters T, Eisenberg D, Duh QY, Curet M. Video self-assessment augments development of videoscopic suturing skill. *J Am Coll Surg*. 2009;209(5):622-625. doi:10.1016/j.jamcollsurg.2009.07.024. Excluded because did not report self-assessment accuracy imrpvoement with video review
7. Dowling S. Clinical evaluation: A comparison of self, self with videotape, peers, and supervisors. *The Clinical Supervisor*. 1984;*2*(3):71-78. Excluded because participants were not fully licensed physicians
8. Neumann M, Hahn C, Horbach T, et al. Score card endoscopy: a multicenter study to evaluate learning curves in 1-week courses using the Erlangen Endo-Trainer. *Endoscopy*. 2003;35(6):515-520. doi:10.1055/s-2003-39670. Excluded because there was no video intervention
9. Wang PZT, Xie WY, Nair S, Dave S, Shatzer J, Chahine S. A Comparison of Guided Video Reflection versus Self-Regulated Learning to Teach Knot Tying to Medical Students: A Pilot Randomized Controlled Trial. *J Surg Educ*. 2020;77(4):805-816. doi:10.1016/j.jsurg.2020.02.014. Excluded because participants who received the intervention were not fully licensed physicians (although resident physicians were enrolled to act as controls for comparison)
10. Tudor GJ, Podolej GS, Willemsen-Dunlap A, et al. The Equivalence of Video Self-review Versus Debriefing After Simulation: Can Faculty Resources Be Reallocated?. *AEM Educ Train*. 2019;4(1):36-42. Published 2019 Aug 12. doi:10.1002/aet2.10372. Excluded because self-assessment accuracy was not measured.
11. Plant JL, Corden M, Mourad M, O'Brien BC, van Schaik SM. Understanding self-assessment as an informed process: residents' use of external information for self-assessment of performance in simulated resuscitations. *Adv Health Sci Educ Theory Pract*. 2013;18(2):181-192. doi:10.1007/s10459-012-9363-2. Excluded because self-assessment accuracy was not measured.
12. Thammasitboon S, Butiu T, Mendoza NL, Gellula M, Noronha PA, Artega, G. A mock code utilizing video feedback: A tool to enhance physicians performance and self-efficacy in pediatric resuscitation. *Pediatric Research.*2002;51(4 Part 2): 70A. <https://eurekamag.com/research/034/297/034297035.php>. Excluded because only published in abstract form.
13. Bull NB, Silverman CD, Bonrath EM. Targeted surgical coaching can improve operative self-assessment ability: A single-blinded nonrandomized trial. *Surgery*. 2020;167(2):308-313. doi:10.1016/j.surg.2019.08.002. Excluded because there was no true baseline of self-assessment accuracy established before introduction of an intervention
