# Supplementary material for: Video-based interventions to improve self-assessment accuracy among physicians: A systematic review
Source: PLoS One. 2023 Jul 13;18(7):e0288474. doi: 10.1371/journal.pone.0288474 (PMC10343035; doi:10.1371/journal.pone.0288474)
Supplement: Attachment — Submitted filename: Rebuttal Letter.doc [file pone.0288474.s005.doc]

February 15, 2023,

Dr. Emily Chenette

Editor-in-chief, PLOS ONE

Dear Dr. Chenette and members of the editorial board:

Thank you for giving us the opportunity to revise and resubmit our manuscript (PONE-D-22-30231) entitled “Video-based interventions to improve self-assessment accuracy among physicians: A systematic review.” We thank the reviewers for their insightful and thoughtful comments. We have addressed all of the reviewers’ queries and have outlined our responses and any corresponding changes below:

**EDITORIAL COMMENTS**

(1) *Please ensure that your manuscript meets PLOS ONE's style requirements, including those for file naming.*

We have addressed all style requirements, including for file naming.

(2) *Thank you for stating the following in the Competing Interests section:
       "Other disclosures: Rishad Khan has received research grants from AbbVie and Ferring Pharmaceuticals and research funding from Pendopharm. Samir C. Grover has received research grants and personal fees from AbbVie and Ferring Pharmaceuticals, personal fees from Takeda, education grants from Janssen, and has equity in Volo Healthcare." Please confirm that this does not alter your adherence to all PLOS ONE policies on sharing data and materials, by including the following statement: "This does not alter our adherence to PLOS ONE policies on sharing data and materials.” Please include your updated Competing Interests statement in your cover letter; we will change the online submission form on your behalf.*

We have added the required statement to the Cover Letter.

(3) *Please include your tables as part of your main manuscript and remove the individual files. Please note that supplementary tables (should remain/ be uploaded) as separate "supporting information" files*

We have included the tables in the body of the manuscript and as separate files as requested.

(4) *Please include captions for your Supporting Information files at the end of your manuscript, and update any in-text citations to match accordingly.*

We have added the appropriate captions.

**REVIEWER #1**

*(1)The manuscript is very well written. Research methodology and selection process can both be clearly understood. Notwithstanding this, this reviewer has to express his doubt about exhaustivity of study selection. The authors state: „We only examined studies of physicians (and not medical students), as we aimed …“. Although the Strudy of Scaffidi et al includes 35-47% of students in each arm. How do the authors support this inclusion?*

We agree that this point requires clarification. Our intention in seeking only to examine studies of physicians was that we wanted to represent a narrower group, as we do not consider medical students to be representative of fully licensed physicians. Nevertheless, the inclusion of the study by Scaffidi et al that has a proportionally significant amount of medical students does call this criterion into question. To clarify this, we have added a qualifier in the Methods that we sought to include studies in which the majority of participants were physicians (pg 7).

*(2) On my opinion the low amount of studies included, assessing very different, heterogeneous medical skills among physicians with different experience levels does not allow to support a general statement about the trained technique as the authors try to support. Since the method evaluation of the different studies does not allow comparison, it seems uncareful to asses a statement about the overall performance of this intervention. The authors describe briefly some effect of experience of the physician in the discussion. Although, this reviewer would suggest putting more effort explaining effect size of each study or the relevance of experience.*

We also agree that there are heterogeneous medical skills among physicians with different experience levels. We have commented on this as a limitation in the Discussion (pg. 14). Additionally, it is true that there are different measures of effects (which we have highlighted already as one of the limitations).

**REVIEWER #2**

(1) *The language needs some revision as the sentences are often very long and therefore difficult to read.*

We have addressed this point by reviewing the manuscript and making changes as appropriate.

(2) *Exclusion criteria of the 988 excluded studies should be mentioned in more detail. Why are so many studies excluded? How many studies were excluded because of the lack of what kind of data?*

We agree that many studies were excluded. Most of these studies, however, were excluded because they failed to properly evaluate self-assessment accuracy with comparison of self-assessment scores to an external observer. We have acknowledged this point in the Results section (pg. 9).

(3) *The characteristics of the interventions (line 236-238) do not add up to 100%.*

We acknowledge that there was an error in the calculation, which is corrected in the Results section (pg. 10). Nevertheless, we still would not expect that the numbers add up to 100% as there is overlap between the interventions. For example, self-video was evaluated in 8 of 9 studies but 3 of the 8 studies also involved benchmark video intervention. The combination of benchmark and self-video interventions was evaluated 3 of 9 studies, while benchmark was evaluated in 4 of 9 studies. Therefore, only 1 study exclusively evaluated the benchmark video intervention. The purpose of this line was to highlight that self-video was the most common video intervention.

(4) *More detailed information about the nine included studies should be added to the study characteristics in details. It would be more informative for the reader to mention the surgical disciplines as well as the number of participants and their expertise not only in the table but also as a sum-up in the text.*

We agree that these details would be beneficial and have added them in the Results (pg. 10).

(5) *It might be interesting to display if there is an under-or overestimation of skills and what disciplines tend towards which direction. The percentage of over- vs. under-estimation of performance in the different studies and study-types would be interesting.*

We agree that these details would be useful to demonstrate and have incorporated the recommendations into the Results (pg 11).

(6) *It should be considered to display and discuss the results of the different study types, e.g. studies for enhancing practical skills and studies for improving verbal/communication skills separately.*

We have provided the requested context in the Results section (pg. 11).

(7) *The identified risks of bias could be explained more clearly in the text. (MS)*

We have explained the risk of bias more clearly in the Results section (pg. 12).

Thank you once again for your consideration of this manuscript. We look forward to your review and comments.

Yours sincerely,

Samir C. Grover, MD, MEd, FRCPC

Division of Gastroenterology

St. Michael’s Hospital
