# Supplementary material for: Video-based interventions to improve self-assessment accuracy among physicians: A systematic review
Source: PLoS One. 2023 Jul 13;18(7):e0288474. doi: 10.1371/journal.pone.0288474 (PMC10343035; doi:10.1371/journal.pone.0288474)
Supplement: Attachment — Submitted filename: Rebuttal Letter.doc [file pone.0288474.s006.doc]

May 29, 2023

Dr. Emily Chenette

Editor-in-chief, PLOS ONE

Dear Dr. Chenette and members of the editorial board:

Thank you for giving us the opportunity to revise and resubmit our manuscript (PONE-D-22-30231) entitled “Video-based interventions to improve self-assessment accuracy among physicians: A systematic review.” We thank the reviewers for their insightful and thoughtful comments. We have addressed all the reviewers’ queries and have outlined our responses and any corresponding changes below:

**ACADEMIC EDITOR COMMENTS**

(1) *In case of small samples, percentages are not recommended to be reported; use instead the ration (e.g. 3/9)*

We agree with this recommendation and have removed the percentages throughout the manuscript.

(2) *Self assessment of what? cooking? physical examination? please be specific.*

We have addressed this comment in the manuscript by specifying in the abstract that we are investigating self-assessment of a physician’s own performance on both procedural and non-procedural tasks in a given speciality of medicine or surgery.

(3) *Please avoid in scientific writing the following expressions "There is/are".*

We have removed the expression in the revised manuscript.

(4) *The aim: any kind of physicians?*

Yes, we were intentionally inclusive for several reasons. First, there is a general paucity of evidence in the first place for interventions aimed at improving self-assessment accuracy. Second, although there is heterogeneity among all physicians based on specialty, practice area and demographics, we conceptualize the practice of self-assessment on procedural tasks as a rather homogeneous activity, owing to its constrained parameters, i.e. assessment of one’s own performance of an external task.

(5) *"limited among physicians and surgeons" please define limited.*

We have clarified this with the term “inaccurate” In the Introduction (pg 4).

(6) *lacking in which way?*

We have clarified that there had not been a systematic evaluation of the literature until our present manuscript.

(7) *An update of PRISMA, namely PRISMA-2020, exists and should be followed.*

An updated and completed PRISMA-2020 checklist had been uploaded with the original manuscript submission. We have provided the updated reference.

(8) *"PHYSICIANS" used in the search string would not retrieve manuscripts with "physician"*

Our search strategy was created by a qualified health research librarian employed by the University of Toronto Gerstein Library. She has assured us that there would not be any manuscripts missing with the use of “PHYSICIANS” as opposed to “physician.”

(9) *The main reason for exclusion at screening is lack of definition of accuracy; this is the main issue of your study because it is well known that such detailed information could not necessary stay in the abstract of the manuscript.*

We kindly refer the Editor to the Definitions section of the Methods (pg 5), wherein we explicitly define what we mean by self-assessment accuracy, which is “direct comparison between an external evaluator and self-assessment that was quantified using formal statistical analysis”. We have clarified this in the Abstract (pg 2).

(10) *Please list the other reasons for exclusion.*

We have clarified the other reasons for exclusions in the Methods (pg 6).

(11) *The reason of exclusions must stay in the prisma flowchart not in supplementary file.*

We have not provided the list of reasons for exclusions, but rather put the full text references excluded.

(12) *"was an assessment tool with prior validity evidence, as used in 4 (44.4%) studies." please provide the references for these studies.*

We have added the relevant references in the Results (pg 10).

(13) *The exclusion of studies in which no clear definition of accuracy was present in the abstract is a major limitation. Furthermore, inclusion of studies written in English is another limitation.*

We have added these two limitations in the Discussion (pp 13-14).

(14) *You include studies with pre and post assessment; it is unclear why this information is reported in Table 1.*

We have removed redundant information in Table 1.

(15) *Please do not duplicate the results in text and tables.*

Initially, our draft manuscript did not duplicate this; we, however, added in the data in Table form at the request of the reviewers.

(16) *In Table 2 it is not clear how the scores were attributed. A solution is to present briefly the instrument in the Material and Methods section.*

We have added in a brief description of the MERSQI tool in the Material and Methods (pg. 8).

**REVIEWER #2**

(1) *The language still needs some revision as some words are used incorrectly and sentences do not make sense. For example:
    347 Quantifying this relationship would advance the objective impact of accurate self-assessment on
    clinical practice.
    63 Self-assessment can be used to identify physicians’ deficiencies to allow for appropriate corrective measures.
    102 Not only can inaccurate self-assessment impair opportunities for growth, but overconfidence stemming from one’s own performances can lead to patient safety concerns.
    Disagreements were resolved by consensus.
    256 In contrast, of the 2 studies in which self-video review was effective, both were from General Surgery and 1 was procedural.*

We have addressed these points by reviewing the manuscript and making changes as appropriate.

(2) *The Conclusions in the Abstract should describe the results of the manuscript in more detail, for example:
    Benchmark video review may enable physicians to improve self-assessment accuracy, especially for those with limited experience in performing a particular clinical skill. Self-video review may be able to improve the self-assessment of more experienced physicians’.*

We appreciate the suggestion and have incorporated this into our abstract conclusion.

(3) *Citing of Literature should be avoided in the Conclusions (omit the sentence in line 342-344 and put it in the right context in the discussion).
    The first sentence of the Conclusions should be omitted as it does not provide a conclusion. Suggestion: Our review highlights the following suggestions for medical education.*

We have addressed this point by reviewing the manuscript and making changes as appropriate.

Thank you once again for your consideration of this manuscript. We look forward to your review and comments.

Yours sincerely,

Samir C. Grover, MD, MEd, FRCPC

Division of Gastroenterology

St. Michael’s Hospital
